# Cancer may accelerate locomotive syndrome and deteriorate quality of life: a single-centre cross-sectional study of locomotive syndrome in cancer patients

**DOI:** 10.1007/s10147-023-02312-2

**Published:** 2023-02-19

**Authors:** Masahiro Hirahata, Jungo Imanishi, Wataru Fujinuma, Satoshi Abe, Takahiro Inui, Naoshi Ogata, Satoshi Iimuro, Retsu Fujita, Kenji Sato, Toru Tokizaki, Taisuke Matsuyama, Hirotaka Kawano

**Affiliations:** 1 Orthopaedic Surgery, Teikyo University School of Medicine, 2-11-1 Kaga, Itabashi-ku, Tokyo, 173-8605 Japan; 2 Rehabilitation Medicine, Teikyo University School of Medicine, 2-11-1 Kaga, Itabashi-ku, Tokyo, 173-8605 Japan; 3 Innovation and Research Support Center, International University of Health and Welfare, 4-1-26, Akasaka, Minato-ku, Tokyo, 107-8402 Japan

**Keywords:** Locomotive syndrome, Cancer, Activities of daily living, Quality of life

## Abstract

**Background:**

Thanks to recent advancement in cancer treatment, an increasing number of cancer patients are expected to live longer with cancer. The ambulatory ability is essential for cancer patients to spend their own independent lives, but locomotive syndrome (LS), a condition of reduced mobility due to impairment of locomotive organs, in cancer patients has been seldom examined.

**Methods:**

This was a single-institutional cross-sectional study. Cancer patients receiving cancer therapy between April 2020 and March 2021 were asked to participate. LS was classified as stage 0–3, and compared with their performance status (PS). Physical component summary (PCS) and mental component summary (MCS) were calculated from the results of Short Form-8. Logistic regression analysis was performed to identify risk factors for LS stage 3.

**Results:**

One hundred and seventy-six cancer patients were included. The rate of LS was 96.0%. That of LS stage 3 was 40.9% and as high as 29.7% even if limited to those with PS 0. The mean PCS and MCS were both inferior to the national averages. PCS decreased as the LS stage advanced. Old age and underweight were revealed as independent risk factors for LS stage 3.

**Conclusions:**

The ratio of LS in cancer patients was extremely high, and the LS stage correlated with physical QOL. Even those with PS 0 can have severe LS; thus, LS can be a sensitive detector of physical disability of cancer patients than PS. The improvement of LS can be a key to the preservation of their ADL and QOL.

**Supplementary Information:**

The online version contains supplementary material available at 10.1007/s10147-023-02312-2.

## Introduction

Cancer is the leading cause of death worldwide, with approximately 10 million deaths reported annually [1]. Its impact is remarkable amongst aged and super-aged societies represented by Japan, the most aged society at present. In 2016, Japan first recorded over 1 million new cancer cases per year [2], with this figure overtaking the number of new births. As cancer therapy has been greatly improving, more and more cancer patients are expected to live longer with disease. Since 2006, when the Cancer Control Act was enacted, Japan has been focussing on “curing” cancer, and has achieved increased life expectancy for cancer patients. At least one cancer centre was designated in each prefecture, and the even distribution of high-quality cancer information and care was sought. The prognosis for cancer patients has certainly improved; the 5-year relative survival rate of any type of cancer in Japan rose from 48.9% for males and 59.0% for females diagnosed in 1993–1996 to 62.0% and 66.9% for those diagnosed in 2009–2011, respectively [3]. In contrast to the improved survival, it is questionable whether cancer patients have been ‘cared’ for appropriately in terms of activities of daily living (ADL) and quality of life (QOL).

The 2006 Cancer Control Act promoted the establishment of a society where cancer patients can live comfortably with human dignity. Mobility can be a key to the preservation of human dignity because human beings, as “social creatures”, need to access to public spaces for social activities. In addition, higher levels of physical activity can reduce the risk of premature all-cause mortality [4]. Although locomotive organs are directly responsible for mobility, little is known regarding reduced mobility due to impairment of locomotive organs in cancer patients.

In 2007, the Japanese Orthopaedic Association (JOA) proposed “locomotive syndrome” (LS). LS was defined as a condition wherein mobility functions are declined due to impairment of locomotive organs [5]. This concept was introduced to increase the awareness of this condition and to reduce an increasing burden of nursing care. Thanks to the campaign of “prevention of LS” promoted by JOA, the importance of preventing LS for longer and healthier life became widely accepted. In 2018, JOA expanded their activities to the field of oncology; they decided the annual activity theme as “LS in cancer patients”.

The purposes of this study were to clarify the degree and ratio of LS in cancer patients, the impact of LS on QOL of cancer patients, and risk factors for LS stage 3 in cancer patients.

## Materials and methods

### Ethical approval

The study protocol was approved by the Institutional Review Board of our institution (19-239, 19-272, 20-007), corresponding to Cohort A, B, and C as below. The patients and/or their families were informed that data from the case would be collected, analysed, and submitted for publication, and consent for their participation was obtained in written form.

### Participants

This was a cross-sectional study on locomotive functions of cancer patients receiving cancer therapy between April 2020 and March 2021. The subjects of three cohorts were asked to participate in this study. The subjects were divided into three cohorts according to the way of recruitment. Cohort A was a group of in-hospital cancer patients receiving cancer therapy and were prescribed in-hospital rehabilitation by the oncologist in charge of each patient. Cohort A consisted of patients undergoing surgery for primary cancer and those with advanced cancer admitted to hospital for non-surgical treatment. Cohort B was a group of cancer patients receiving outpatient chemotherapy. Cohort C was a group of cancer patients referred to the department of orthopaedic surgery due to orthopaedic problems including bone metastasis and non-neoplastic orthopaedic issues such as osteoarthritis and lumbar spinal canal stenosis, regardless of whether inpatient or outpatient.

### Index tests

LS was evaluated using three LS risk tests: the stand-up test, the two-step test, and the 25-question Geriatric Locomotive Function Scale (GLFS-25) [6, 7]. The risk levels for LS were classified as stage 0–3 according to the scores as below, applying the concept of “LS stage 3”, which was introduced by JOA in 2020 [8].

#### Stand-up test

Stools of four different heights (40, 30, 20, and 10 cm) were used. The subjects stood up from a sitting position using both legs or one leg. The scoring system included nine performance scores: 0 (inability to stand); 1, 2, 3, and 4 (stand using both legs from a height of 40, 30, 20, and 10 cm, respectively); and 5, 6, 7, and 8 (stand using one leg from a height of 40, 30, 20, and 10 cm, respectively). Scores 3–4, 2, and 0–1 were classified as stages 1, 2, and 3, respectively.

#### Two-step test

Subjects took two steps with the longest possible stride, and the stride length for the two steps was measured. The test score was calculated as the length of the two steps divided by the height of each subject. Scores ≥ 1.1 to < 1.3, ≥ 0.9 to < 1.1, and < 0.9 were classified as stages 1, 2, and 3, respectively.

#### GLFS-25

The subjects were asked to fill out the questionnaire for assessing their physical status and living circumstances, with each of the 25 items scored on a range of 0–4. The total score (ranging from 0 to 100) was used for the analyses. Scores 7–15, 16–23, and 24–100 were classified as stages 1, 2, and 3, respectively.

#### Total assessment (overall stage)

The total assessment was determined based on the results of the aforementioned three tests. Of the corresponding stages, the stage to which mobility function had decreased the most was adopted as the overall stage.

### Association between Eastern Cooperative Oncology Group performance status (ECOG PS) and LS

At registration, ECOG PS was scored for each patient [9]. The number of subjects at each PS score and each overall LS stage was counted and listed in the form of correspondence table.

### Summarised scores of Short Form-8 (SF-8)

SF-8 was used for the evaluation of QOL. SF-8 questionnaire translated into Japanese consists of 8 domains. Physical component summary (PCS) and mental component summary (MCS) were calculated from the answers towards 4 questions for each summary, using a published algorithm. Higher scores indicate better QOL. National averages for scores including PCS and MCS are available [10, 11].

### Possible risk factors for LS stage 3

Old age (≧ 65 years old), female, obesity (≧ 25.0 kg/m^2^), underweight (< 18.5 kg/m^2^), metastatic bone tumour, chemotherapy, hormone therapy, and low back or joint pain were expected to be factors associated with LS stage 3 based on previous studies and clinical perspectives. Yamada et al. reported that all three test scores gradually decreased amongst young-to-middle-aged individuals and rapidly decreased in individuals aged over 65 years in a cross-sectional nationwide study in Japan [12]. They also addressed the difference between genders in the speed of decrease in the two-step test results amongst middle-aged individuals. Yoshimura et al. reported that most subjects with sarcopenia and/or frailty also had LS [13]. As sarcopenia and/or frailty are associated with obesity and/or underweight, respectively, these two factors were thought to be possible risk factors for LS stage 3. Tsuji et al. reported that patients with chronic pain had a significant rate of LS [14]. Although there were no reports about the relationship between LS and bone tumour, chemotherapy or hormone therapy, these were thought to possibly affect locomotive functions and were thus selected as possible risk factors. The cancer stage was considered inappropriate as a factor because it has different meanings depending on the type of cancer and is frequently being revised.

### Statistical analysis

All data are expressed as means. The distribution of each LS stage for each LS risk test and the total LS stage were compiled. Logistic regression analysis was performed assuming that the factors for LS stage 3 were old age, female, obesity, underweight, metastatic bone tumour, chemotherapy, hormone therapy, and low back or joint pain.

The analysis was conducted using SAS 9.4 software (SAS Institute Inc., Cary, NC, USA). The level of significance was set at 5%.

## Results

One hundred and eighty-two patients agreed to participate in the present study. Later, one patient withdrew one’s consent and five patients could not complete the GLFS-25 and were excluded, so that a total of 176 patients were analysed. Table 1 shows patient demographics and clinical characteristics for the 82 female and 94 male patients (mean age 70 [range 30–89] years) analysed in this study.

**Table 1:** Patient demographics and clinical characteristics

	Total (*n* = 176)	Percentage
Age, yrs	70 (30–89)	
Old age > 65	103	58.5
Sex		
Female	82	46.6
Male	94	53.4
Body mass index, kg/m^2^	23 (15—39)	
Underweight < 18.5	22	12.5
Obesity > 25.0	63	35.8
Cohort		
A: In-hospital rehabilitation	85	48.3
B: Outpatient chemotherapy	40	22.7
C: Consultation	51	29.0
Primary tumour		
Breast	42	23.9
Gastric	20	11.4
Lung	15	8.5
Prostate	12	6.8
Colon	10	5.7
Others	77	43.8
Bone metastasis	49	27.8
Chemotherapy	87	49.4
Hormone therapy	34	19.3
Low back or joint pain	105	59.7

**Fig. 1:**
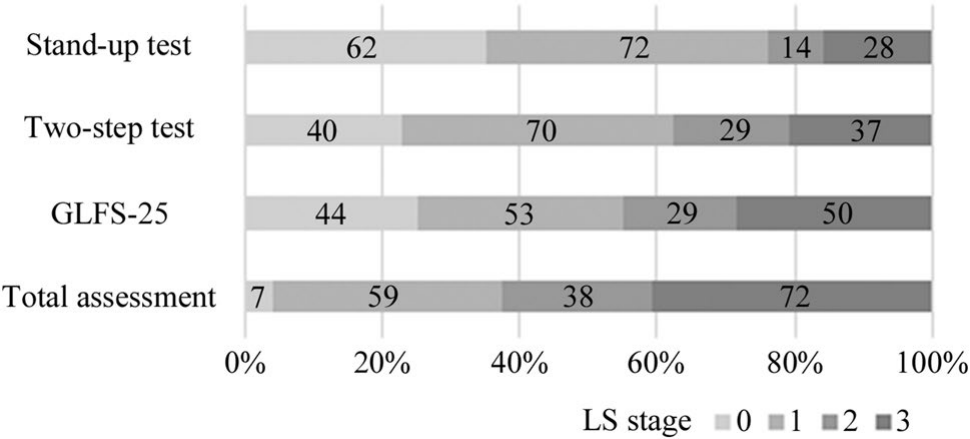
The distribution of locomotive syndrome stage in three tests and total assessment. The number of cases classified as each stage was written in each square

**Fig. 2:**
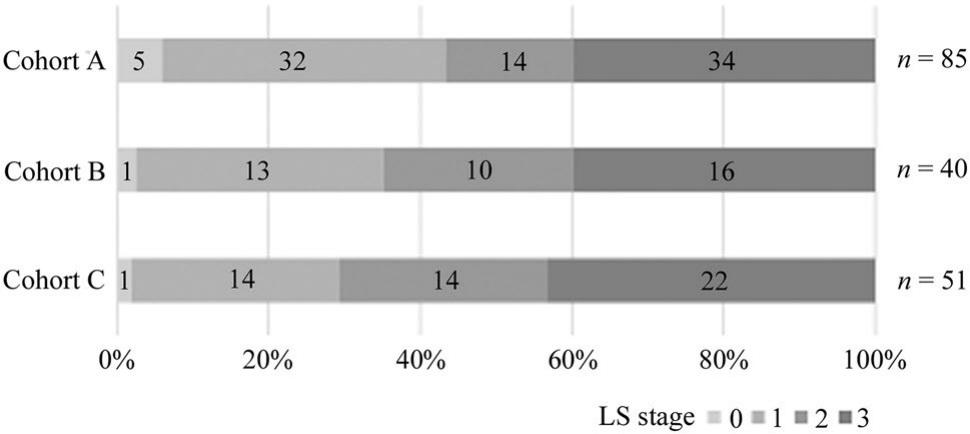
The distribution of locomotive syndrome stage in total assessment according to the cohort. The number of cases classified as each stage was written in each square

The rate of overall LS was 96.0%, with 33.5% for stage 1, 21.6% for stage 2, and 40.9% for stage 3. According to the type of test, the rate of LS for the stand-up test was 64.8%, with 40.9% for stage 1, 8.0% for stage 2, and 15.9% for stage 3. That for the two-step test was 77.3%, with 39.8% for stage 1, 16.5% for stage 2, and 21.0% for stage 3. That for the GLFS-25 was 75.0%, with 30.1% for stage 1, 16.5% for stage 2, and 28.4% for stage 3 (Fig. 1). As per the cohort, the rate of overall LS was 94.1% for Cohort A, 97.5% for Cohort B, and 98.1% for Cohort C (Fig. 2).

Table 2 shows the association between ECOG PS and overall LS stage. PS was 0–1 amongst the majority of the subjects (166/176, 94.3%); however, LS stage was 3 in as much as 29.7% and 60.4% of those with PS 0 and 1, respectively.

**Table 2:** The correspondence table between the stage of locomotive syndrome and performance status

	Stage of locomotive syndrome				Total
	0	1	2	3	
PS 0	7	51	25	35	118
PS 1	0	8	11	29	48
PS 2	0	0	1	7	8
PS 3	0	0	1	1	2
PS 4	0	0	0	0	0
Total	7	59	38	72	176

**Fig. 3:**
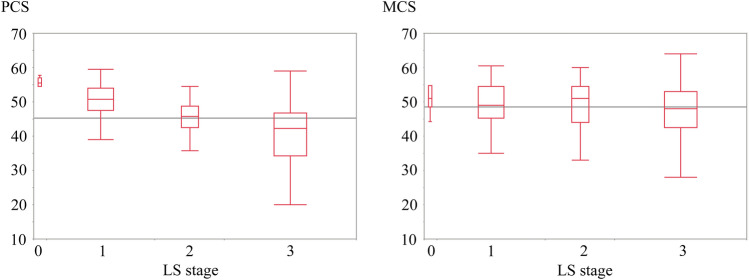
The distribution of the physical component summary (PCS) and mental component summary (MCS) of Short Form-8 (SF-8), according to the locomotive syndrome stage. The upper and lower ends of the box indicate the 75th percentile and 25th percentile, respectively. The horizontal lines in each box indicate the 50th percentile, the median of each stage. Two horizontal lines across LS stages indicate the PCS and MCS averages of all the subjects, 45.3 and 48.5, respectively

The mean PCS and MCS were 45.3 and 48.5, both of which were inferior to the national averages of 48.9 and 49.3, respectively. Figure 3 shows the distribution of PCS and MCS according to the LS stage in the form of boxplot. The mean PCS decreased as the LS stage advanced, being 54.8 for non-LS (stage 0), 50.2 for stage 1, 44.8 for stage 2, and 40.6 for stage 3. In contrast, the mean MCS stayed flat between stages 0 and 2.

Logistic regression analysis revealed that old age and underweight were independent risk factors for LS stage 3, with their odds ratios being 2.7 (95% CI 1.3–5.6, *P* = 0.01) and 3.3 (95% CI 1.2–9.5, *P* = 0.03), respectively (Table 3).

**Table 3:** Logistic regression analysis assuming the factors for locomotive syndrome stage 3

	OR	95% CI	*P* value
**Old age**	**2.7**	**1.3–5.6**	**0.01**
**Underweight**	**3.3**	**1.2–9.5**	**0.03**
Female sex	0.6	0.3–1.3	0.24
Chemotherapy	1.3	0.6–2.5	0.49
Hormone therapy	0.8	0.3–2.0	0.61
Bone metastasis	1.2	0.5–2.6	0.66
Obesity	0.9	0.4–1.8	0.71
Low back or joint pain	1.0	0.5–2.0	0.93

Bold indicates significant difference (*p* < 0.05)

*OR* odds ratio, *CI* confidence interval

## Discussion

The current study surveyed the degree of LS in cancer patients in a cross-sectional manner, and revealed that the rate of LS in cancer patients was incredibly high at 96.0% in total and 40.9% for stage 3. This study is the first to highlight the high prevalence of LS stage 3, severely impaired locomotive function in cancer patients, and address the importance of the recognition of and action towards resolving such issues. The interpretation of such high percentage of LS requires some care as this was a cross-sectional study with no comparison. A prospective study of population-based cohorts conducted by Yoshimura et al. reported that the rate of LS was 81.0% [13]. The mean age of the subjects in the study was 72 years, whereas that in this study was 70 years old. Although the direct comparison of 81.0% and 96.0% is inappropriate, it can be said that cancer patients receiving cancer therapy are probably more likely to develop LS than the general population. According to a recent comparison study using propensity score, LS rate of cancer patients was 89% and significantly higher than that of non-cancer patients [15]. In their study, the concept of LS stage 3 was not applied, hence the rate of LS stage 3 was unknown. In contrast, our study revealed that locomotive function of more than 40% of the cancer patients was graded as LS stage 3, i.e. they showed progressive decline in mobility function and limited social participation. Yoshimura et al. reported that 11.6% of participants from general population, with the mean age of 65.6 years, were at LS stage 3, and LS stage 3 significantly increased that the risk of disability and mortality [16]. Our result warrants a prompt response, including the elucidation of its pathology and appropriate treatment intervention, so as to remedy this issue.

Attention should be paid to the relationship between PS and LS in cancer patients. PS is a simple measure of physical function and general condition of cancer patients, and is widely used to determine the administration of cancer therapy [9]. PS 0–1 or 0–2 is often regarded as a minimal requirement for administration of intensive chemotherapy. As such, oncologists may have the impression that a patient with PS 0 is certainly in overall good condition. However, in the current study, as much as 29.7% of the subjects with PS 0 suffered LS stage 3. This result indicates that cancer patients can suffer severe LS even if their PS is judged as fair. To our best knowledge, the relationship between LS and PS in cancer patients has never been reported. The authors propose that LS risk tests can be an early detector of physical dysfunction of cancer patients.

Amongst the three LS risk tests, the GLFS-25 can be the most practical to detect LS in cancer patients. In the current study, the rate of LS stage 3 was 28.4% in the GLFS-25, which was higher than 15.9% in the stand-up test and 21.0% in the two-step test. Kobayashi et al. reported that the categorisation using the GLFS-25 was more sensitive than that using the other two, both of which were physical tests [17]. Since then, several studies have determined LS stages from the GLFS-25 only. In addition, GLFS-25 can be safer than the other two because the both physical tests involve fall risk.

This study has revealed that cancer can affect not only ADL but also QOL of cancer patients, and is the first study to show the correlation between the LS stage and QOL of cancer patients. As illustrated by Fig. 3, physical QOL deteriorated as the LS stage advanced, and even mental QOL also slightly decreased at LS stage 3 in this study. The results mean that locomotive functions probably affect physical QOL of cancer patients even from earlier LS stage, and can deteriorate mental QOL if LS advances.

LS in cancer patients can be classified into three types, as proposed by the “Cancer LOCOMO” working group in Japan [18]. Type 1 is LS caused by the cancer itself. The direct causes of impairment in locomotive functions can be pain, paralysis, and fractures due to bone and soft tissue tumours, regardless of whether they are primary or metastatic. Type 2 is LS caused by cancer treatment. Examples are disuse syndrome associated with resting for chemotherapy, osteoporosis associated with hormone therapy and steroid use, and chemotherapy-induced peripheral neuropathy. Type 3 is LS caused by the progression of coexisting orthopaedic diseases, such as lumbar spinal canal stenosis and osteoarthritis. All the three factors, cancer itself, cancer therapy, and coexisting orthopaedic diseases, are thought to have negative impacts on locomotive functions, but the pathology of LS in cancer patients is not so simple that every case can be classified into a single category. Two or three types can coexist; for instance, a patient can be exhausted by repetitive chemotherapy, suffer symptomatic spinal metastasis, and have pseudogout at the same time. For LS in cancer patients, the possibility of each of the aforementioned three types should be considered when deciding an appropriate treatment; especially, unnecessary instructions to rest, which can worsen disuse syndrome, should be avoided.

Old age and underweight were revealed as independent risk factors for LS stage 3 in the current study. “Old age” is as expected because locomotive function generally declines with age. In contrast, the association between “underweight” and LS stage 3 is interesting. Bodyweight is thought to correlate with skeletal muscle mass [19], and underweight can be a prominent clinical feature in cachexia [20]. As cancer patients often develop cachexia, our result may demonstrate a strong association between LS stage 3 and cachexia. However, the relationship between underweight and LS may be bidirectional as disuse syndromes can also exist in underweight cancer patients. As disuse syndrome is reversible, rehabilitation and self-exercise may be effective to prevent and improve LS.

By contrast to “old age” and “underweight”, there was no significant association between LS stage 3 and bone metastasis in this study. Prior to this study, it was anticipated that the existence of bone metastasis would be an independent risk factor for severe LS, but it was not. This finding may just be the result of the small sample size; however, is seems to indicate that the cause of severe LS is not merely the existence of bone metastases but can be different, supporting the concept of three types of LS as mentioned above.

The current study had several limitations. First, the sample size was relatively small, and some risk factors for LS stage 3 may have been overlooked. Second, selection bias existed as we could not recruit all possible cancer patients for this study. For example, inoperative cases due to poor general conditions were not included, whereas surgical cases with lower risk for peri-operative complications may have been more often omitted from rehabilitation prescription, in the current study. Third, due to the COVID-19 pandemic, the situations regarding both patients and medical practitioners may have differed from those in ordinary times [21]. Lastly, the natural course of LS in cancer patients remains to be clarified due to the cross-sectional nature of this study. A similar study with a larger number of subjects and an observational study of the subjects to clarify the natural course of LS in cancer patients are required.

In conclusion, the rate of LS in cancer patients, particularly stage 3, which prevents social participation, was incredibly high and correlated with decreased QOL in this study. The result of nearly 30% of the cancer patients with PS 0 suffering LS stage 3 indicates that LS risk tests can be an early detector of physical disability, and LS of cancer patients can be a target for intervention. Further research is mandatory to understand this disease concept in more detail, and to clarify how and when to intervene so as to improve it.

## Supplementary Information

Below is the link to the electronic supplementary material.

Supplementary file1 (DOCX 23 KB)
