# Supplementary material for: Cancer may accelerate locomotive syndrome and deteriorate quality of life: a single-centre cross-sectional study of locomotive syndrome in cancer patients
Source: Int J Clin Oncol. 2023 Feb 19;28(4):603–9. doi: 10.1007/s10147-023-02312-2 (PMC9939082; doi:10.1007/s10147-023-02312-2)
Supplement: Supplementary file 1 — Supplementary file1 (DOCX 23 KB) [file 10147_2023_2312_MOESM1_ESM.docx]

**Supplementary Data**

**Supplementary Table 1. The relationship between the existence of bone metastasis and locomotive syndrome stage 3.**

| Cohort | Bone metastasis | Locomotive syndrome stage 3 | | P value |
| --- | --- | --- | --- | --- |
|  |  | (+) | (-) |  |
| A | (+) | 7 | 6 | 0.26 |
|  | (-) | 27 | 45 |  |
| B | (+) | 6 | 4 | 0.14 |
|  | (-) | 10 | 20 |  |
| C | (+) | 8 | 18 | 0.07 |
|  | (-) | 14 | 11 |  |
| Total |  | 72 | 104 |  |
